# Methicillin-resistant *Staphylococcus aureus* without borders: USA300 in Cuba

**DOI:** 10.1186/1753-6561-5-S6-P172

**Published:** 2011-06-29

**Authors:** J Hopman, G Toraño Peraza, F Espinosa, CH Klaassen, DM Velázquez, JF Meis, A Voss

**Affiliations:** 1 Medical Microbiology, Radboud University Nijmegen Medical Centre, Nijmegen, Netherlands; 2 Bacteriology-Mycology, Pedro Kourí Institute, Havana, Cuba; 3 Microbiology, Hermanos Ameijeiras Hospital, Havana, Cuba; 4 Medical Microbiology and Infectious Diseases, Canisius-Wilhelmina Hospital, Nijmegen, Netherlands

## Introduction / objectives

Methicillin-resistant *Staphylococcus aureus* (MRSA) is an increasing problem in the Americas and the Caribbean including Cuba. Recently, MRSA isolates are emerging as significant pathogens in the community. In the USA, the most prevalent community associated (CA-)MRSA clone is USA300 (ST8, spa type 008). Little is known about the molecular epidemiology of MRSA in some of the Caribbean countries. In this study we aim to investigate the molecular epidemiology of MRSA isolates from 4 major Cuban hospitals.

## Methods

During a 3 months period in 2008 all clinical isolates suspected to be MRSA were prospectively collected. Three major Cuban hospitals and the national reference centre for infectious diseases participated in the study. Oxacillin susceptibility testing was performed in Cuba. Further examinations were done in the Netherlands, including Panton-Valentine leukocidin(PVL), genes *luk*S-*luk*V, mecA and Spa-typing.

## Results

From the 56 suspected Staphylococcus isolates, 38 were confirmed to be MRSA. Spa typing identified 5 different spa-types. In decreasing frequency we found Spa-type t149, t008, t037, t4088 and t2029, in respectively 22, 8, 6, 1 and 1 isolates. Only the eight t008 isolates were PVL positive.

## Conclusion

Here we report the first molecular typing results of MRSA isolates from Cuba. The predominant clone was the Spa-type 149, followed by CA-MRSA USA300. We conclude that an economic and political embargo is a weak infection control measurement to contain the spreading of a potentially harmful pathogen.

## Disclosure of interest

None declared.

